# The Latest Crazy Man

**Published:** 1868-11

**Authors:** 


### THE LATEST CRAZY MAN.

Some person has written a book "On the Principles of Organic Life," and what is more remarkable than this, has found a respectable publisher in London—who maintains that all the great virtues to be found in man, are due to the retention in his body of the fæces, he says:

"Viewing all these matters from first to last, the only conclusion physiology can come to, debasing as it may appear, yet nevertheless true, is, that man owes all his thoughts and aspirations, and all the useful efforts of his great and exalted mind to his *fæces*."

Comment is unnecessary!

